# Modification of Physico-Chemical and Biological Characteristics of Polymethylmethacrylate with Amorphous Carbon Nanoparticles for Counteracting Healthcare-Associated Infections

**DOI:** 10.3390/jfb17010005

**Published:** 2025-12-21

**Authors:** Sergey V. Gudkov, Dmitriy A. Serov, Ruslan M. Sarimov, Vasiliy S. Novikov, Maksim Moskovskiy, Maksim B. Rebezov, Mikhail V. Dubinin, Konstantin V. Sergienko, Mikhail A. Sevostyanov, Fatikh M. Yanbaev, Maxim E. Astashev, Maria V. Vedunova

**Affiliations:** 1 Prokhorov General Physics Institute of the Russian Academy of Sciences, Vavilov St. 38, 119991 Moscow, Russia; s_makariy@rambler.ru (S.V.G.); rusa@kapella.gpi.ru (R.M.S.); vasiliy1992@gmail.com (V.S.N.); dubinin1989@gmail.com (M.V.D.); astashev@yandex.ru (M.E.A.); mvedunova@yandex.ru (M.V.V.); 2 Department of Fundamental Sciences, Bauman Moscow State Technical University, 2ya Baumanskaya St. 5/1, 105005 Moscow, Russia; 3 Federal Scientific Agroengineering Center VIM, 109428 Moscow, Russia; maxmoskovsky74@yandex.ru; 4 Department of Scientific Research, Gorbatov Research Center for Food Systems, Talalikhin St. 26, 109316 Moscow, Russia; rebezov@yandex.ru; 5 Faculty of Biotechnology and Food Engineering, Ural State Agrarian University, Karl Liebknecht St. 42, 620075 Yekaterinburg, Russia; 6 Department of Biochemistry, Cell Biology and Microbiology, Mari State University, pl. Lenina 1, 424001 Yoshkar-Ola, Russia; 7 Baikov Institute of Metallurgy and Materials Science, Russian Academy of Sciences (IMET RAS), Leninsky Prospekt 49, 119334 Moscow, Russia; shulf@yandex.ru (K.V.S.); cmakp@mail.ru (M.A.S.); 8 Federal Research Center “Kazan Scientific Center of the Russian Academy of Sciences”, Lobachevskogo St. 2/31, 420088 Kazan, Russia; aurum_fr@mail.ru; 9 Institute of Biology and Biomedicine, Lobachevsky State University of Nizhny Novgorod Institute, Gagarin av. 23, 603105 Nizhny Novgorod, Russia

**Keywords:** polymethyl methacrylate-like resin, MSLA, amorphous carbon nanoparticles, polymer composite, antibacterial and non-toxic to mammalian cells

## Abstract

Composite materials based on polymethylmethacrylate (PMMA) and carbon nanoparticles are used in aviation, construction, medical and other fields of activity. Carbon nanotubes and carbon nano-dots are mainly used as carbon nanoparticles. Both carbon nanotube and carbon nano-dots are difficult to obtain materials with considerable cost. Amorphous carbon nanoparticles, on the contrary, are easy to obtain and have a low cost. The purpose of this work is to study the physico-chemical and biological characteristics of polymethylmethacrylate modified with amorphous carbon nanoparticles. Laser ablation was used to obtain the nanoparticles. Dynamic light scattering, measurement of the electrokinetic potential, TEM, AFM, and Raman microscopy are used to characterize nanoparticles. FTIR, MIM, AFM, UV-visual diagnostics, ROS tests, and biopolymer regeneration tests were used to analyze the combined sensors. The bacteriostatic effect was evaluated using turbodimetry, and the antibacterial effect was evaluated using precision cytofluorometry. Mammalian cells were examined using fluorescence microscopy. Carbon nanoparticles (CNPs) have been obtained and characterized. A protocol has been developed for the introduction of CNPs into photolithographic resin. Printed samples of complex geometry. It is shown that the printed samples are amenable to polishing, have pro-oxidant properties, and are able to prevent damage to biopolymers. Printed samples inhibit the development of bacteria and cause loss of viability. At the same time, the printed samples do not affect the development of mammalian cells. The obtained resins based on PMMA with CNPs can potentially serve as the basis for the creation of non-toxic materials in biomedicine and pharmacology.

## 1. Introduction

The incorporation of nanoparticles into a polymer matrix provides a pathway to new material properties [1]. The new properties of such a composite system are based on the interaction between the nanoparticles and the polymer matrix [2]. Commonly reported property enhancements include mechanical, thermal, chemical, and biological characteristics [3]. For instance, the addition of nanoparticles to polymer typically leads to an increase in strength and yield point due to the formation of a strong adhesive bond between the polymer and the nanofiller [4]. An increase in the thermal stability of nanocomposites is also frequently reported [5]. Sometimes, change in the crystallization rate is observed, as nanoparticles act as nucleation sites, affecting the number and size of the formed crystals [6]. Typically, the crystallization rate increases at low nanofiller concentration, while further increase in nanofiller concentration retards the crystallization process [7]. In some cases, the addition of nanoparticles to polymers improves the resistance of nanocomposites to aggressive environments [8] and slows down the processes of thermo-oxidative degradation [9]. Quite often, the incorporation of nanoparticles alters the biological properties of the materials [10], typically by changing the level of toxicity towards biological entities [11]. Alterations in the microrelief of the composites can also influence the success of biofilm formation or cell adhesion on the surfaces [12].

The modification of polymers for additive manufacturing is particularly relevant [13]. The layer-by-layer fabrication process enables the imparting of different properties to different parts of an object [14]. For instance, unmanned aerial vehicle propellers or marine vessel propellers require different mechanical properties at the hub and at the tip of the blades [15]. Antibacterial properties are often needed not throughout the entire volume, but only in the surface layer [16]. Currently, one of the most common and in-demand polymers for additive manufacturing is polymethylmethacrylate (PMMA) and its various formulations [17]. PMMA often exhibits high transparency and low absorption of electromagnetic radiation in the visible spectrum [18]. Most PMMA formulations are dielectrics [19], possess competitive mechanical properties [20], and demonstrate high durability for outdoor applications [21]. Furthermore, PMMA products are insoluble in common solvents [22]. Despite these excellent operational properties, PMMA has significant drawbacks; for example, many bacteria and fungi can grow and proliferate on its surface [23].

Metal and metal oxides nanoparticles are commonly used as nanoadditives for plastics [24]. Most such solutions are effective; however, these nanoparticles often aggregate within the hydrophobic matrix, which prevents their uniform distribution in the polymer matrix [25]. Furthermore, metal nanoparticles tend to release metal ions into the surrounding medium. This can reduce the biocompatibility of the nanocomposites and undesirably increase the electrical conductivity of the environment in contact with the material [26]. An alternative solution is the use of carbon nanoparticles. It has previously been shown that the inclusion of carbon nanotubes in PMMA prevents the adhesion of microorganisms [27]. The addition of graphene nanoplatelets to polymethyl methacrylate improves the mechanical and thermal properties of the composite [28]. The addition of chemically modified carbon nanotubes, fullerenes, and graphene oxide to PMMA made it possible to obtain fire-retardant composites [29]. Composites based on one-dimensional carbon nanofibers with PMMA and other polymers significantly reduce the intensity of microwave radiation [30]. To create high-dielectric materials, polymethyl methacrylate is doped with carbon nanodots [31]. PMMA threads reinforced with carbon nanotubes have significantly better mechanical properties [32]. The inclusion of multiwall carbon nanotubes in PMMA-based bone cement improves its cytocompatibility and osseointegration [33]. It was found that PMMA-based composite material containing multiwall carbon nanotubes with a prolonged release of active substances has antimicrobial properties [34]. As can be seen from the above literature and specialized review articles [35,36], several classes of carbon nanoparticles are mainly used to create composites: fullerenes, carbon nanotubes, graphene, while amorphous carbon is used very rarely [37]. At the same time, among all these allotropic nanoforms of carbon, it is nanosized amorphous carbon that is inexpensive and suitable for industrial production [38]. A wide range of amorphous carbon-based nanoparticles has been developed to date. Many of these nanoparticles can interact with various biomacromolecules [39], exhibit outstanding redox activity [40], and possess membrane-tropic properties [41]. Amorphous carbon also exhibits effective sorbent properties at the macro level [42]. It should be noted that composite materials exhibit different effects depending on the shape and chemical nature of the nanoparticles used. The most pronounced effect is observed upon the introduction of nanoparticles with an extended and geometrically complex shape. The aim of this work was to synthesize complex-shaped amorphous carbon nanoparticles, develop a method for their incorporation into PMMA, and study the physico-chemical and biological properties of products made from this composite material.

## 2. Materials and Methods

### 2.1. Synthesis of Carbon Nanoparticles

Carbon nanoparticles (CNPs) were synthesized via laser ablation of a high-purity graphite target (purity 99.99%) in deionized water. The target was placed at the bottom of a glass flow reactor, with the thickness of the deionized water layer above the target surface maintained at no more than 3 mm. The total volume of deionized water in the system was approximately 100 ml. Deionized water was circulated through the reactor using a peristaltic pump at a flow rate of about 50 mL/min. A P-Mark TT 100 fiber ytterbium laser (Pokkels, Moscow, Russia) was used, operating at a wavelength of 1064 nm, pulse repetition rate of 35 kHz, pulse duration of 200 ns, and pulse energy of 1.5 mJ. The laser beam was focused onto the surface of the bulk graphite target. Beam scanning across the target was achieved using an LScanH galvanometric optical scanning system (Ateko-TM, Moscow, Russia) with an F-Theta lens (focal length 90 mm). The beam was scanned at a speed of 3 m/s along straight lines filling a rectangular area of 20 × 30 mm, with a line spacing of 20 µm.

### 2.2. Characterization of CNPs

The concentration of nanoparticles and their hydrodynamic radius, as well as the ζ-potential, were determined in aqueous colloids using the Malvern Zetasizer Ultra (Malvern Panalytical Ltd., Malvern, Great Britain). The spectra of aqueous colloids of nanoparticles were studied using a Cintra 4040 spectrophotometer (GBC Scientific Equipment Pty Ltd., Keysborough, VIC, Australia). Microimages of nanoparticles were obtained using the Libra 200 FE HR TEM (Carl Zeiss, Jena, Germany). The size of the CNPs was determined using an atomic force microscopy system for morphology analysis (NT-MDT, Zelenograd, Russia). Raman spectra of CNPs were recorded using a Senterra II confocal Raman microscope (Bruker Optics, Billerica, Massachusetts, USA) with 180° scattering geometry. The microscope was equipped with a 20× objective (NA = 0.4). An excitation wavelength of 532 nm was used, with a spectral resolution of 4 cm^−1^ and a laser power of 14 mW at the sample surface.

### 2.3. Addition of CNPs to Photolithographic Resin

The solvent phase of the CNP colloid solution was exchanged from water to acetone. The aqueous CNP colloid solution was centrifuged using a 3-16KL rotor centrifuge (Sigma Laborzentrifugen GmbH, Petershütte, Germany) (60 min, 7000 rpm). The supernatant was decanted and replaced with an equal volume of pure acetone. To facilitate the resuspension of nanoparticles from the bottom of the centrifuge tube into acetone, the tube was subjected sequentially to: (1) ultrasonication (40 kHz, 22 °C, 3 min); (2) vortex mixing (shaker, 2000 rpm, 5 min). This procedure was repeated three times. The resulting CNP colloid solution was stored in a sealed glass vial. Prior to use, the CNP colloid solution in acetone was mixed with Dental Clear PRO lithographic resin (Harz Labs, Mytishchi, Russia) to achieve final nanoparticle concentrations of 0.001%, 0.01%, and 0.1% by weight. After mixing, the resin with nanoparticles was subjected to mechanical agitation (shaker, 2000 rpm, 5 min) followed by ultrasonication (40 kHz, 3 min).

### 2.4. Additive Manufacturing of Composite Material Samples

Samples were fabricated using a Saturn 3 Ultra 12K MSLA printer (Elegoo, Shenzhen, China) from photolithographic resins containing 0.001%, 0.01%, and 0.1% CNPs or without CNPs. The printed samples underwent post-processing. First, they were rinsed in isopropanol (99.9%) for 6 min, then immersed in a fresh portion of alcohol and subjected to ultrasonication for 6 min. Subsequently, the samples were dried at room temperature. After drying, glycerin was applied to the sample surfaces. The glycerin-coated samples were treated in a UW-02 curing unit (Creality3D, Shenzhen, China) for 30 min. This was followed by another ultrasonication step and drying. At the final stage, the samples were exposed to heat at 80 °C for 30 min under normal pressure. The post-processed samples were stored in sealed containers at room temperature.

### 2.5. Methods for Characterizing Composite Materials

The micro- and nanostructure of the printed sample surfaces was investigated using an atomic force microscopy system for morphology analysis NT-MDT (LLC, Zelenograd, Russia). Studies of nanoparticle-polymer matrix interactions were performed using an IR-8000 FTIR (SAS LLC, Krasnoyarsk, Russia) and Cintra 4040 (GBC Scientific Equipment Pty Ltd., Victoria, Australia) spectrometers. The change in the phase shift in laser radiation, which provides information about the distribution of CNPs inside the polymer, was analyzed using a MIM-321 modulation interference microscope (Amphora Laboratories, Moscow, Russia). To study the release of nanoparticles from the polymer matrix, samples containing and not containing nanoparticles were placed in deionized water for 3 days. Each sample was in a separate 20 mL vial (Glastechnik Grafenroda, Geratal, Germany). After that, the probability of breakdown by laser radiation in water was studied (wavelength of 1064 nm, pulse repetition rate of 10 kHz, pulse duration of 4 ns, and pulse energy of 2 mJ). Tensile tests were evaluated using a WDW-5S universal testing machine (Hongtuo, Binzhou, China) according to ASTM D638-22 standard. To carry out thermogravimetric analysis, a MOM Q-1550 D derivatograph was used; the analysis was carried out in air at a heating rate of 10 deg/min.

### 2.6. Measurement of Hydrogen Peroxide Concentration

The concentration of H_2_O_2_ in the aqueous environment was determined by the enhanced chemiluminescence method. Chemiluminescence detection was carried out using a highly sensitive “Biotoks-7A-USE” chemiluminometer (Engineering Center-Ecology, Moscow, Russia). The chemiluminescent reaction mixture was prepared in Tris-HCl buffer containing luminol, p-iodophenol, and horseradish peroxidase. The study utilized sample plates measuring 1 × 1 × 0.05 cm. The plates were placed in antistatic vials filled with 20 mL of deionized water. The vials were incubated at 40 °C for 180 min. The method used allows for the detection of 0.1 nM hydrogen peroxide. Experimental details have been published previously [43].

### 2.7. Measurement of Hydroxyl Radicals

Hydroxyl radicals were quantified using a fluorimetric method based on the formation of 7-hydroxycoumarin-3-carboxylic acid from the reaction between hydroxyl radicals and coumarin-3-carboxylic acid. Test samples in the form of plates (10 × 10 × 0.5 mm) were placed in 20 cm^3^ polypropylene vials filled with an aqueous solution of coumarin-3-carboxylic acid. The vials were incubated at 80 °C for 120 min, after which the pH of the medium was raised to 8.5 and measurements were taken. The resulting fluorescent product—7-hydroxycoumarin-3-carboxylic acid—was detected using a JASCO 8300 spectrofluorometer at excitation and emission wavelengths of 400 nm and 450 nm, respectively. Experimental details have been published previously [44].

### 2.8. Quantitative Determination of 8-Oxoguanine in DNA

The amount of 8-oxoguanine in DNA was determined by enzyme-linked immunosorbent assay (ELISA) using monoclonal antibodies. Experiments utilized sample plates measuring 1 × 1 × 0.05 cm. The samples were added to an aqueous DNA solution (10 mL) and incubated for 3 h at 40 °C. Primary antibodies against 8-oxoguanine were applied at a dilution of 1:2000, and secondary antibodies at a dilution of 1:1000. Incubation with secondary antibodies was performed on a shaker for 1.5 h at 37 °C. Measurements were conducted using a Feyond-A400 (Allsheng, Hangzhou, China) plate photometer at wavelength of 405 nm. Experimental details have been published previously [43].

### 2.9. Quantitative Determination of Long-Lived Reactive Protein Species

The experiments utilized sample plates measuring 1 × 1 × 0.05 cm. The samples were added to an aqueous BSA colloid (10 mL, 0.1%) and incubated for 2 h at 40 °C. After incubation, the aqueous BSA colloid was kept in darkness for 0.5 h at room temperature, after which measurements were performed. The amount of long-lived reactive protein species was assessed using the method of induced chemiluminescence of protein colloids. Luminescence detection was carried out with highly sensitive Biotox-7A chemiluminometer (Engineering Center—Ecology, Moscow, Russia). All experimental details have been described previously [45].

### 2.10. Sterilization of Samples Before Biological Tests

Before conducting biological tests, all samples of composite materials were sterilized. To prevent thermal deformation of the polymer matrix, the samples were pre-processed in drying cabinet at temperature of 50 °C for 24 h. This procedure made it possible to remove residual volatile components (for example, unreacted monomers) and adsorbed moisture, preventing further deformation under high-temperature exposure. After drying, samples from various experimental groups were placed in individual 100 mm glass Petri dishes, labeled, wrapped in foil, packed in a kraft bag for SteriT sterilization (Vinar, Moscow, Russia) and placed in a dry-heat cabinet for 180 min at 120 °C. After complete cooling, the packages were opened in a laminar flow hood with aseptic measures immediately before the experiments. The effectiveness of sterilization was confirmed both by changing the color of the chemical indicator on the package to brown, and by negative sterility control results (incubation of samples in nutrient broth at 37 °C for 7 days).

### 2.11. Dynamics of E. coli Broth Culture Reproduction

In the experiments, samples with a diameter of 10 mm and thickness of 0.5 mm were used. The samples were sterilized and placed into wells of a 24-well plate. Then, 1 mL of LB nutrient medium containing *E. coli* at a concentration slightly below 10^6^ CFU/mL was added to each well. The plate without a lid was placed in a plate photometer-incubator. Measurements were taken every hour for 24 h at a constant temperature of 37 °C with shaking once per hour. Sterile broth without bacteria was used as a control. All experimental details have been described previously [46].

### 2.12. Evaluation of Antibacterial Activity by Flow Cytofluorimetry

Flow cytometry was performed using a Longcyte cytofluorimeter (Challenbio, Beijing, China). Preparation and cultivation of *E. coli* were carried out according to the method described in Section 2.10. Cultures were stained with 4 µM propidium iodide (PI) from Lumiprobe (Maryland, USA, USA) dissolved in 1 mL of phosphate-buffered saline (PBS). For staining, cultures were incubated with PI for 1 h in darkness. Prior to flow cytometry, bacterial cultures were thoroughly resuspended and transferred to sterile 1.5 mL Expell microcentrifuge capp tubes (AHN, Nordhausen, Germany). PI fluorescence intensity was assessed by measuring both side scatter (SS) and forward scatter (FS) (excitation 535 nm, emission 617 n m). Bacterial concentration in the suspension was determined by automated recalculation of event counts per 1 mL of suspension. The percentage of nonviable microorganisms was determined by the fluorescence intensity of PI. All experimental details have been described previously [47].

### 2.13. Cytotoxicity Assessment on Eukaryotic Cells

The biocompatibility of samples printed from photolithographic resins, both with and without CNPs, was studied using a culture of human spleen fibroblasts (HSF) (ATCC line No. PCS-201-012). HSF cultivation was performed in cultural medium DMEM/F12 with FBS (10%), L-Glu (2 mM), streptomycin (25 µg/mL) and penicillin (25 U/mL). The human spleen fibroblasts (HSF) were cultured for 3 days in a CO_2_ incubator. Cell viability was assessed by fluorescence microscopy using the fluorescent dyes Hoechst 33342 (for nuclear staining), Rhodamine (for mitochondrial visualization), and propidium iodide (PI) (for detecting dead cells). Cell visualization and imaging were performed using a Leica DMI 4000B microscopic system. The obtained microphotographs were processed using ImageJ software v 1.54f. The technical details of the experiment are given in [48].

### 2.14. Statistical Data Analysis and Visualization

The results on the graphs are presented as the mean values. In some cases, in the form of mean and standard errors of mean. The number of independent repetitions is at least three. By independent repetition, we mean the result of an experiment on samples manufactured de novo.

## 3. Results

**Figure 1:**
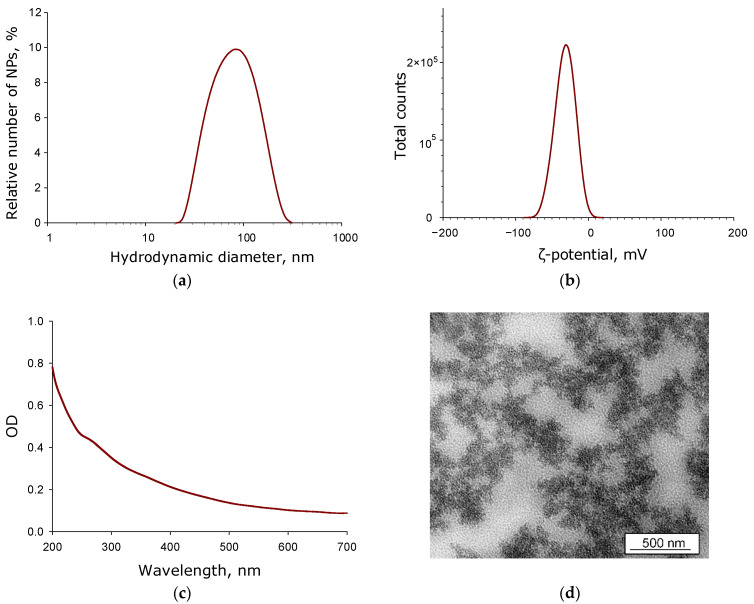
Characteristics of the physicochemical properties of CNPs obtained by laser ablation in the aqueous colloid solution: nanoparticle size distribution (**a**), distribution of the ζ-potential (**b**) and absorption spectrum (**c**). Transmission electron microscopy micrograph of the CNPs sample (scale bar 500 nm) (**d**).

The size distribution of CNPs in an aqueous colloid solution was investigated by dynamic light scattering (DLS) (Figure 1a). The size distribution of CNPs was shown to be monomodal, with one pronounced maximum. A notably broad full width at half maximum was observed in the distribution. This is typically observed when the size distribution contains two dominant, yet closely sized, fractions. Broadening of the distribution can also occur when the particles have a complex shape. The average hydrodynamic diameter of the CNPs was approximately 90 nm, with sizes ranging from 20 to 300 nm. The full width at half maximum of the distribution corresponded to the range of hydrodynamic diameters from 40 to 200 nm.

The distribution of CNPs in the aqueous colloid solution by zeta potential was also investigated using DLS (Figure 1b). The zeta potential distribution of CNPs was shown to be monomodal, with a single pronounced maximum. The average zeta potential of the CNPs in the aqueous colloid was approximately −31 mV. At the same time, the colloid solution contained CNPs with zeta potential values ranging from +10 to −70 mV. The full width at half maximum of the distribution covered zeta potentials from −10 to −50 mV.

The nanoparticle morphology was investigated by transmission electron microscopy (Figure 1d). It should be noted that carbon exhibits relatively weak contrast in electron microscopy, which hinders the visualization of individual CNP smaller than a few tens of nanometers. A fairly concentrated sample of CNPs appears as an array of complex-shaped grains with varying contrast. The size of single grain can be estimated as several tens of nanometers.

**Figure 2:**
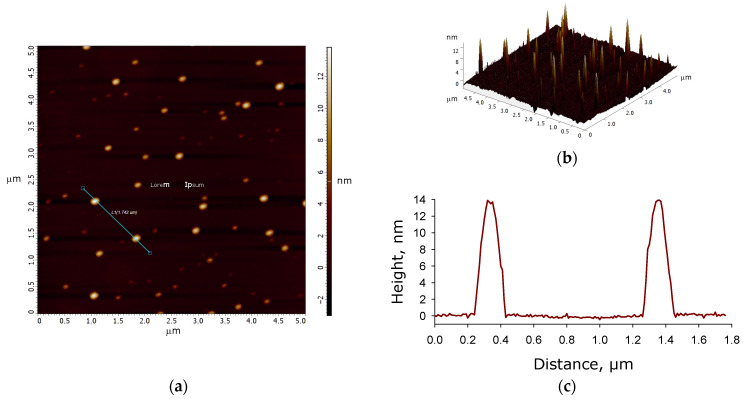
Atomic force microscopy study of CNPs: height map indicated by a teal line (**a**), 3D reconstruction of the height map (**b**), and the height profile (**c**).

To clarify the dimensions of the CNPs, they were examined in dry form using atomic force microscopy (Figure 2). The nanoparticle density on the substrate is approximately 2.5 CNPs/µm^2^. A teal line on the height map (Figure 2a) marks the direction along which the height profile was measured (Figure 2b, bottom). The particle width at half maximum is approximately 150 nm. It should be noted that this represents the size of the two largest CNPs on the height map (Figure 2a). The 3D reconstruction of the height map (Figure 2b, top) suggests that the vast majority of CNPs fall within the size range of 30 to 150 nm.

**Figure 3:**
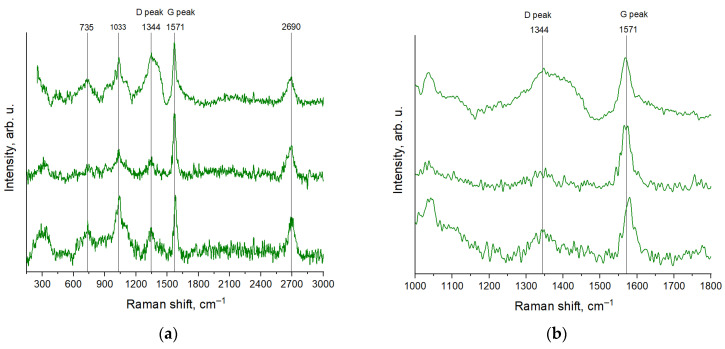
Raman spectra of CNPs recorded with excitation wavelength of 532 nm in different points on the sample in the region 250–3000 cm^−1^ (**a**) and in the region 1000–1800 cm^−1^ (**b**).

Figure 3a shows the Raman spectra of CNPs recorded at several points on the sample. Bands are observed at approximately 735, 1033, 1344 (D band), 1571 (G band), and 2690 cm^−1^. The bands around 735 and 1030 cm^−1^ are typical for carbon powders. The band around 2690 cm^−1^—the second-order mode of the 1344 cm^−1^ band—exhibits low intensity in the studied samples compared to the D and G bands, which is characteristic of amorphous carbon. The spectra indicate sample heterogeneity. The intensity ratio of the D to G bands ranges from 0.3 to 0.8 and varies across different sample areas (Figure 3b). This ratio, combined with the large width of the G band, indicates a high density of defects and the amorphous nature of the material.

CNPs were synthesized via laser ablation of a graphite target in water. It is known that lithographic resins are immiscible with water; moreover, the presence of even trace amounts of water in lithographic resins is unacceptable. On the other hand, acetone is highly soluble in lithographic resins. Therefore, the water in the colloid was replaced with acetone. To achieve this, the aqueous CNP colloid solution was centrifuged for 20 min at 27,000 rpm. The CNPs settled at the bottom of the tube, while the water remained in the supernatant. The supernatant was then removed, and the CNPs deposited at the bottom were resuspended in pure acetone under ultrasonication. It was shown that after replacing water with acetone, a small amount of water (less than 1%) still remained in the colloid. When such colloid was added to the lithographic resin, the printed objects often contained defects. To eliminate the residual water, the solvent exchange procedure was repeated with pure acetone. After this second exchange, defects in the printed objects became rare. After performing the solvent exchange with pure acetone a third time, a colloid containing no significant amount of water was obtained. This colloid, when mixed with the lithographic resin, allowed for the printing of defect-free objects. Various macroscopic components, including round plates, complex parts, and mesh-like scaffolds, were fabricated using this composite lithographic resin containing CNPs.

**Figure 4:**
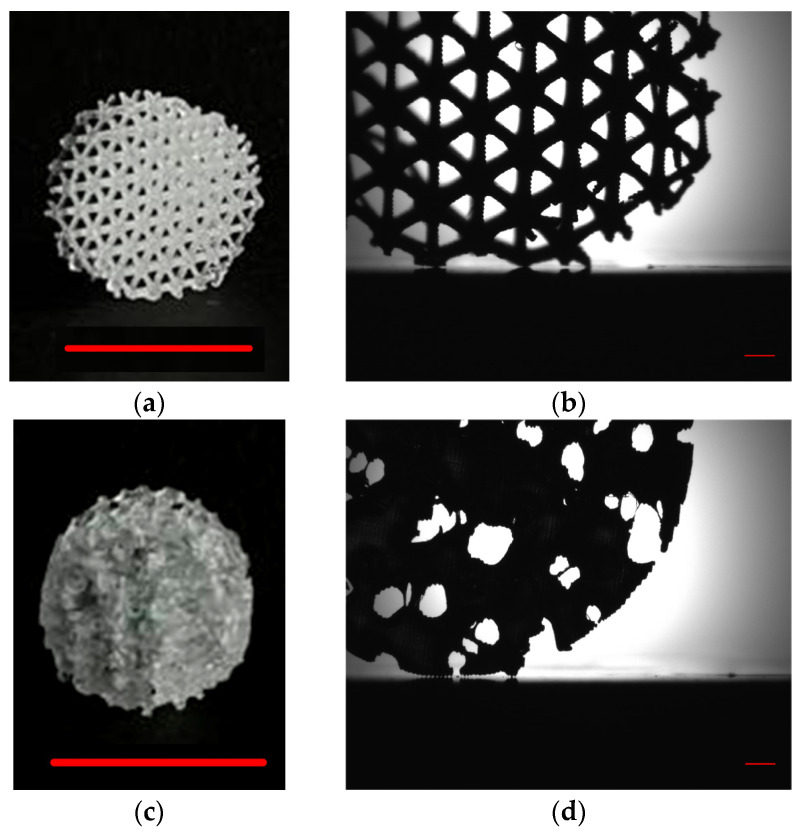
Images of samples printed from the composite lithographic resin containing 0.1% carbon nanoparticles (PMMA/CNPs). Printed scaffold: macroscopic photograph (**a**) and shadow micrograph (**b**). Printed sponge: macroscopic photograph (**c**) and shadow micrograph (**d**). Scale bar in the macroscopic photographs: 10 mm; scale bar (red lines in bottom center/right) in the shadow micrographs: 1 mm.

The printing technology we employed enables the fabrication of both optically transparent plates and macroscopic components from PMMA/CNPs material. Figure 4a,b show an example of an ordered, geometrically precise structure (scaffold), while Figure 4c,d presents an example of a disordered structure that visually distorts space. The addition of CNPs to the lithographic resin did not degrade the spatial resolution of fine details and edges. The maximum resolution we achieved matched that of the masked stereolithography (MSLA) printer and was approximately 50 µm.

**Figure 5:**
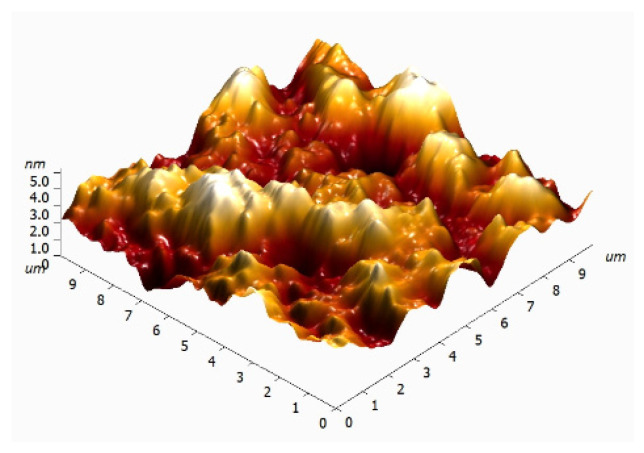
Reconstruction of the surface of samples made of composite lithographic resin containing 0.1% carbon nanoparticles (PMMA/CNPs) after polishing.

Round plates fabricated from lithographic resins with and without CNPs underwent thorough grinding and polishing until they exhibited a clear (undistorted) reflection of objects in visible light. The surfaces of the samples were examined using atomic force microscopy. After grinding and polishing, no cracks, craters, or cavities were detected on the surface of any plates (pure resin or resin with CNP concentrations up to 0.1%). A typical three-dimensional surface profile of a plate made from photolithographic resin containing 0.1% CNPs is shown in Figure 5. It was established that the maximum height variation over an area of approximately 100 µm^2^ does not exceed 10 nm.

**Figure 6:**
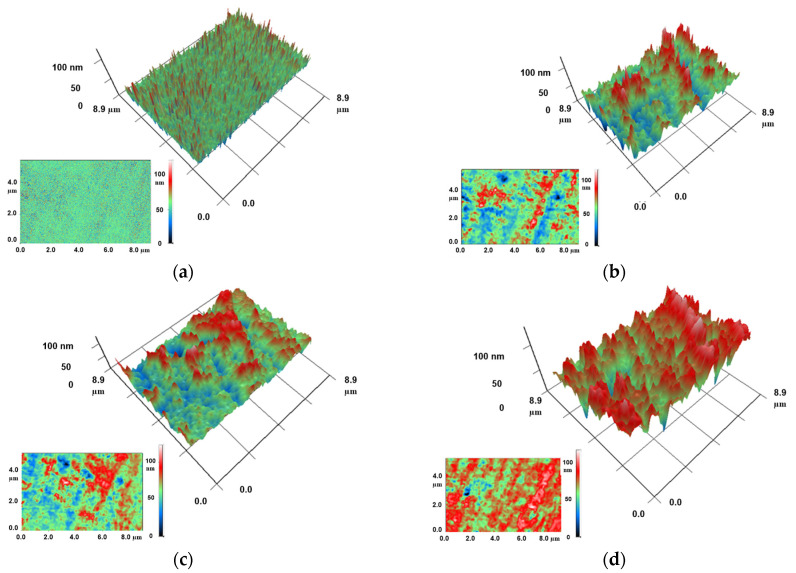
Phase shift profile maps in samples fabricated from photolithographic resin (PMMA) without CNPs (**a**) and resins containing CNPs at concentrations of 0.001% (**b**), 0.01% (**c**), and 0.1% (**d**), obtained using modulation interference microscopy. The profile maps are presented as 3D reconstructions of material sections measuring 8.9 × 8.9 μm, along with the primary data (2D) used for their construction (insets at bottom left). Color represents the phase shift in transmitted radiation (red—maximum value, blue—minimum).

The internal structure of photolithographic resin-based samples with CNPs was investigated using modulation interference microscopy (Figure 6). This technique enables visualization of internal defects in transparent media and can detect the spatial distribution of inclusions with a refractive index differing from that of the base material. In samples without CNPs (0%), no distinct regions with uniform phase shift were detected. The phase shift in each examined pixel was largely random (Figure 6a). In samples containing 0.001% CNPs, small regions (typically ≤0.5 µm^2^) with similar phase shift values became observable (Figure 6b). At 0.01% CNP concentration, regions of similar phase shift reached approximately 1 µm^2^ (Figure 6c). Finally, in samples with 0.1% CNPs, regions of several µm^2^ with comparable phase shift were clearly detectable (Figure 6d).

**Figure 7:**
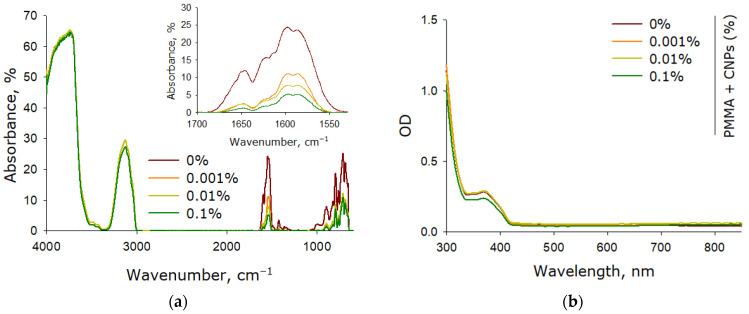
Spectral characteristics of samples fabricated from photolithographic resin (PMMA) without CNPs and resins containing CNPs at concentrations of 0.001%, 0.01%, and 0.1%. FTIR absorption spectra of the samples (**a**): inset shows an enlarged spectral region with bands associated with carbon-carbon double bonds (C=C). UV-Vis absorption spectra of the samples (**b**).

The optical properties of the fabricated plates were investigated using FTIR and UV-Vis spectroscopy. FTIR spectroscopy allows for the study of material composition based on their interaction with infrared radiation (Figure 7a). It was shown that the samples exhibit bands characteristic of organic compounds. In photopolymerizing polymers, the most significant spectral changes are observed in the bands associated with carbon-carbon double bonds (C=C). These bands provide information about the quantity of unreacted resin monomers remaining after photopolymerization. It was established that the absorption intensity in the C=C spectral region of samples without CNPs is approximately 25% of the pre-polymerization level. The introduction of CNPs into the samples leads to a decrease in absorption in the region associated with carbon double bonds. At a CNP concentration of 0.1%, the absorption in the spectral region associated with C=C bonds decreases nearly fivefold compared to the sample without CNPs and amounts to approximately 5% of the pre-polymerization level.

The optical properties of the obtained samples in the ultraviolet and visible range were studied using a differential double-beam UV-Vis spectrometer (Figure 7b). It was found that both samples containing CNPs and those without nanoadditives exhibit one absorption maximum (375 nm) and one absorption minimum (335 nm). The presence of CNPs in the samples, even at a concentration of 0.1%, does not introduce any distinctive features to the absorption spectrum.

**Figure 8:**
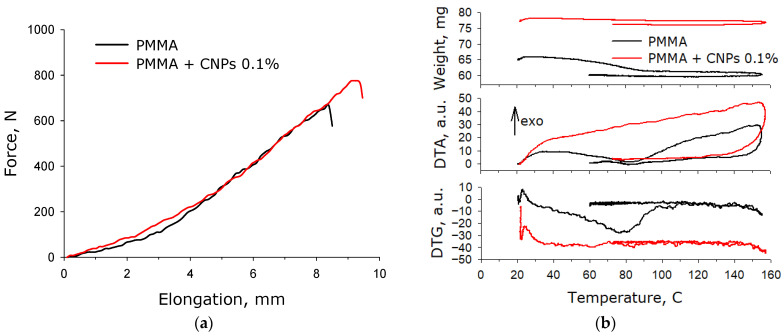
Mechanical (**a**) and thermal (**b**) properties of photolithographic resin (PMMA) without CNPs and resins containing CNPs at concentrations of 0.1%.

The mechanical properties of photopolymer resin samples containing and without nanoparticles at a concentration of 0.1% were studied (Figure 8a). After applying a force to the photolithographic resin sample containing no nanoparticles, elongation of the sample is observed. The curve exhibits two sections that can be linearized. In the first section (up to 110 N), approximately 35 N must be applied to elongate the sample by 1 cm. In the second section, approximately 100 N must be applied to elongate the sample by 1 cm. The sample ruptured at an elongation of 8.4 mm under a force of 660 N. After applying a force to the photolithographic resin sample containing nanoparticles at a concentration of 0.1%, elongation of the sample is observed. The curve exhibits two sections that can be linearized. In the first section (up to 220 N), to extend the sample by 1 cm, it is necessary to apply approximately 55 N. In the second section, to extend the sample by 1 cm, it is necessary to apply approximately 105 N. The sample ruptured at an elongation of 9.2 mm with a force of 770 N.

Thermogravimetric analysis of photolithographic resin (PMMA) with-out CNPs and resins containing CNPs at concentrations of 0.1% was carried out (Figure 8b upper). It was shown that a sample made from a photolithographic resin without nanoparticles loses about 4% of its weight when heated to 90 °C. When heated from 90 to 150 °C, there was no significant change in weight. When cooled from 150 to 60 °C, there was no change in weight either. A sample made from a photolithography resin with CNPs loses just over 1% of its weight when heated to 90 °C. When heated from 90 to 150 °C, there was no significant change in weight. When cooled from 150 to 60 °C, there was no change in weight either.

Using differential thermal analysis (DTA), it was shown that when a sample of photolithographic resin without nanoparticles is heated to 50 °C, an exothermic process is observed (Figure 8b in the middle). When heated from 50 to 90 °C, an endothermic process is observed. When heated from 90 to 150 °C, an exothermic process is observed again. During cooling, an endothermic process occurs. When a sample of photolithographic resin with CNPs is heated, an exothermic process is observed over the entire temperature range; when cooled, an endothermic process is observed.

A differential thermogravimetric analysis was carried out (Figure 8b lower).; it was shown that when a sample made of photolithographic resin without nanoparticles is heated, the rate of change in the weight of the sample changes; when cooled, the rate does not change. For the photolithographic resin with CNPs, no significant changes in the rates of weight change were recorded. It should be noted that the first peak on DTG is an instrument artifact associated with the beginning of heating.

**Figure 9:**
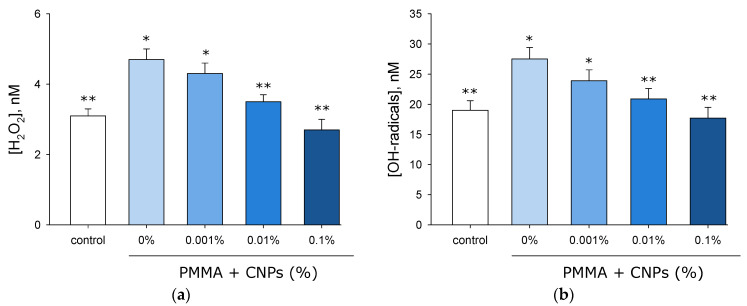
Effects of samples fabricated from photolithographic resin (PMMA) with and without CNPs on the generation of hydrogen peroxide (H_2_O_2_) (**a**) and hydroxyl radicals (^•^OH) (**b**). Data are presented as the mean values ± SEM (*n* = 3). *—significantly different from the control group (*p* < 0.05); **—significantly different from the group of samples without CNPs (0%) (*p* < 0.05).

The effect of polymer samples with and without CNPs on hydrogen peroxide formation in water was investigated using the enhanced chemiluminescence method (Figure 9a). It was shown that without samples (control), approximately 3 nM of hydrogen peroxide forms in water. When a sample without CNPs is added to water, about 5 nM of hydrogen peroxide is generated. In the presence of a sample with 0.001% CNPs, approximately the same amount of hydrogen peroxide forms as with the sample without CNPs. In the presence of a sample with 0.01% CNPs, less than 4 nM forms, which is 20% lower than in the group of samples without CNPs. In the presence of a sample with 0.1% CNPs, less than 3 nM forms, which is slightly lower than in the control group.

The influence of samples fabricated from lithographic resin with and without CNPs on hydroxyl radical generation was assessed using fluorometry with a CCA fluorescent probe (Figure 9b). It was shown that the control group exhibits hydroxyl radical generation at approximately 20 nM. When a sample without CNPs is added to water, about 27 nM of hydroxyl radicals are produced. In the presence of sample with 0.001% CNPs, a slightly lower amount of hydroxyl radicals forms compared to samples without CNPs. In the presence of a sample with 0.01% CNPs, slightly more than 20 nM of hydroxyl radicals are generated, which is comparable to the control group values and lower than in the group of samples without CNPs. In the presence of a sample with 0.1% CNPs, the same amount of hydroxyl radicals is produced as in the control group.

**Figure 10:**
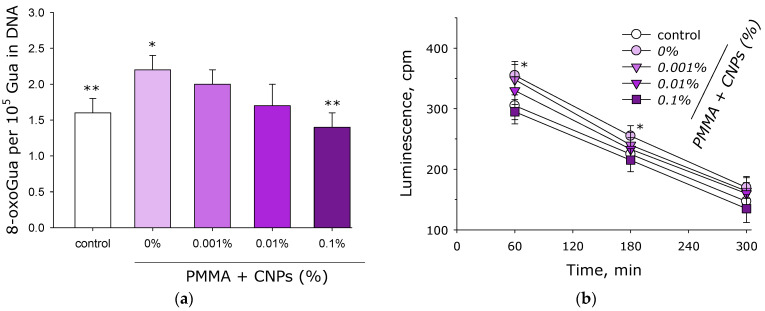
Effects of samples fabricated from photolithographic resin (PMMA) with and without CNPs on the generation of 8-oxoguanine in DNA (**a**) and long-lived reactive protein species (**b**). Data are presented as the mean values ± SEM (*n* = 3). *—significantly different from the control group (*p* < 0.05); **—significantly different from the group of samples without CNPs (0%) (*p* < 0.05).

The effect of samples with and without CNPs on the formation of 8-oxoguanine in DNA in vitro was investigated using an enzyme-linked immunosorbent assay (ELISA) with antibodies specific to 8-oxoguanine (Figure 10a). Under control conditions, approximately 1.5 molecules of 8-oxoguanine per 10^5^ guanines in DNA are formed. When a sample without CNPs is added to DNA, slightly more than two molecules of 8-oxoguanine per 10^5^ guanines in DNA are generated. With the addition of a sample containing 0.001% CNPs, about two molecules of 8-oxoguanine per 10^5^ guanines in DNA are formed, which is comparable to samples without CNPs. In the presence of a sample with 0.01% CNPs, significantly fewer than two molecules of 8-oxoguanine per 10^5^ guanines in DNA are produced, comparable to the control group values and lower than in the group of samples without CNPs. In the presence of a sample with 0.1% CNPs, the same number of 8-oxoguanine molecules per 10^5^ guanines in DNA is formed as in the control group.

The formation of long-lived reactive protein species induced by samples with and without CNPs was studied using the method of induced luminescence (Figure 10b). It was shown that in the absence of samples (control), the formation of long-lived reactive protein species is observed, with an initial luminescence intensity of approximately 300 cpm. When a sample fabricated from lithographic resin without nanoparticles is added to the protein colloid, the initial luminescence intensity increases to approximately 350 cpm. In the presence of samples containing 0.001% CNPs, numerically similar results were obtained. In the presence of samples containing 0.01% CNPs, the luminescence values slightly exceed those in the control group. In the presence of samples containing 0.1% CNPs, luminescence values slightly lower than those in the control group are recorded. Interestingly, the average half-life of long-lived reactive protein species in all studied groups is approximately 5 h.

**Figure 11:**
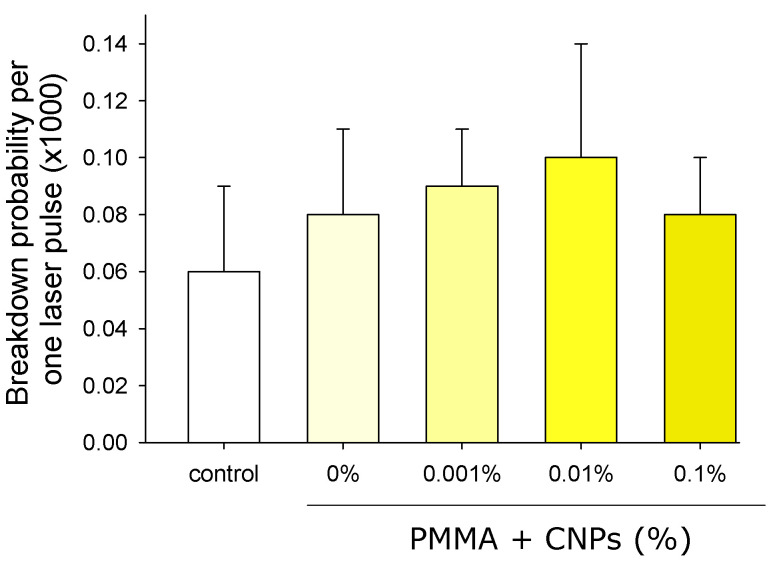
The probability of optical breakdown of a liquid containing porous PMMA blanks with different CNPs contents for 3 days. The presented data is multiplied by 1000. Deionized water was used as a control.

To study the release of nanoparticles from the polymer into the surrounding liquid, porous blanks containing and not containing nanoparticles were placed in water for 3 days. After that, the blanks were removed, and attempts were made to cause a laser breakdown in the remaining liquid (Figure 11). It is shown that the probability of breakdown in all the studied groups is close to 10^−4^. The introduction of blanks into the water did not affect the probability of optical breakdown.

**Figure 12:**
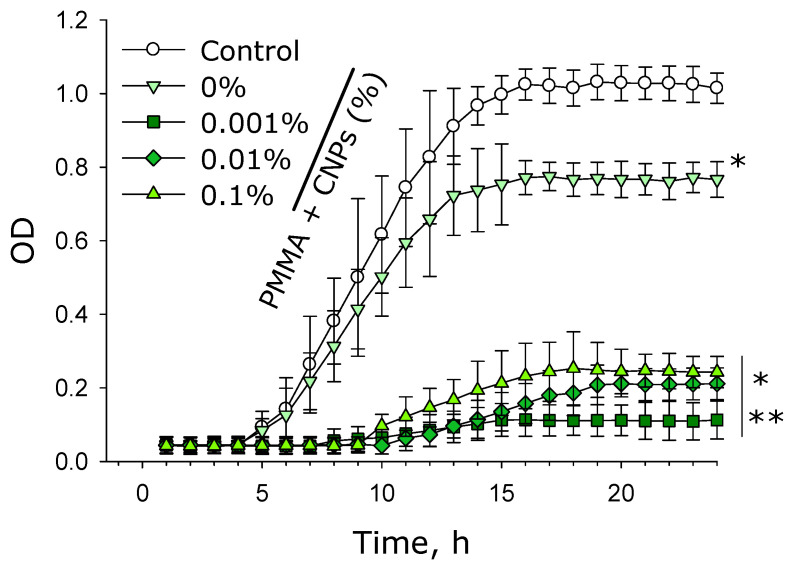
Growth curves of *E. coli* suspension cultures cultivated in the presence of photolithographic resin (PMMA) samples with and without CNPs. Data are presented as the mean values ± standard error of the mean (*n* = 3). *—significantly different from the control group (*p* < 0.05); **—significantly different from the group of samples without CNPs (0%) (*p* < 0.05).

The effect of samples fabricated from photolithographic resin with and without CNPs on the growth and development of suspension cultures of *E. coli* was investigated (Figure 12). It was shown that in the control group, the lag phase duration was approximately 4 h. The logarithmic phase was observed from 4 to 16 h inclusive. During the stationary phase, the optical density of the culture fluid was about 1.0, and the number of microorganisms increased approximately 20-fold from the beginning of the experiment.

In the presence of samples without CNPs, the lag phase duration and logarithmic phase remained unchanged compared to the control group. However, the number of bacterial cells during the stationary phase was 20–25% lower than in the control. In the presence of samples containing 0.001% CNPs, the lag phase lasted 8 h, and the logarithmic phase ended by the 18th hour from the start of the experiment. In the presence of samples with 0.01% CNPs, the lag phase lasted 10 h, and the logarithmic phase ended by the 19th hour. Furthermore, during the stationary phase, the bacterial count was 70–75% lower compared to the control. In the presence of samples fabricated from lithographic resin with 0.1% CNPs, no statistically significant increase in bacterial numbers was observed until the 14th hour relative to the start of the experiment. The logarithmic phase was not pronounced, and during the stationary phase, the number of microorganisms was only twice that at the beginning of the experiment (Figure 12).

**Figure 13:**
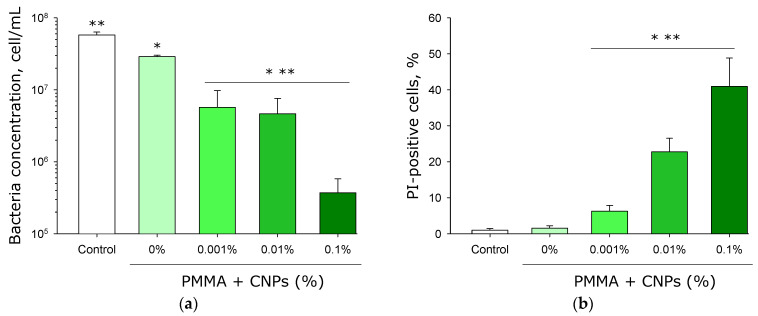
Effects of samples fabricated from photolithographic resin (PMMA) with and without CNPs on the growth and viability of *E. coli* bacteria. Data were obtained by flow cytometry. Concentration of *E. coli* bacteria (**a**) and proportion of dead cells (**b**) after 24 h of cultivation. Data are presented as the mean values ± standard deviation (*n* = 3). *—significantly different from the control group (without samples) (*p* < 0.05); **—significantly different from the group of samples without CNPs (0%) (*p* < 0.05).

Flow cytometric histograms showing the distribution of bacterial cells by geometric mean intensity of PI fluorescence were obtained. Analysis of these histograms enabled numerical determination of the concentration of viable *E. coli* cells and the proportion of non-viable *E. coli* cells. It was shown that when *E. coli* is cultured in medium without samples (control), approximately 5.7 × 10^7^ cells/mL are detected (Figure 13a). When samples without CNPs are introduced, approximately 2.9 × 10^7^ cells/mL are detected in the culture medium. In the presence of samples from lithographic resin (PMMA) containing 0.001% and 0.01% CNPs, about 5 × 10^6^ cells/mL are observed in the culture medium, i.e., one order of magnitude lower than in the control medium and five times lower than in the presence of the samples from lithographic resin without CNPs. In the presence of samples from lithographic resin containing 0.1% CNPs, 3.7 × 10^5^ cells/mL are observed in the culture medium. Thus, in the presence of the PMMA-based sample with 0.1% CNPs, the bacterial count in the culture medium is less than 1% of that in the control.

It was shown that the proportion of non-viable bacteria grown in the control medium or in the presence of samples without CNPs is minimal (Figure 13b). In the presence of samples from lithographic resin containing 0.001% CNPs, the proportion of non-viable bacterial cells significantly exceeds 5%. In the presence of samples with 0.01% CNPs, the proportion of non-viable bacterial cells exceeds 20%, while in the presence of samples with 0.1% CNPs, it exceeds 40%.

**Figure 14:**
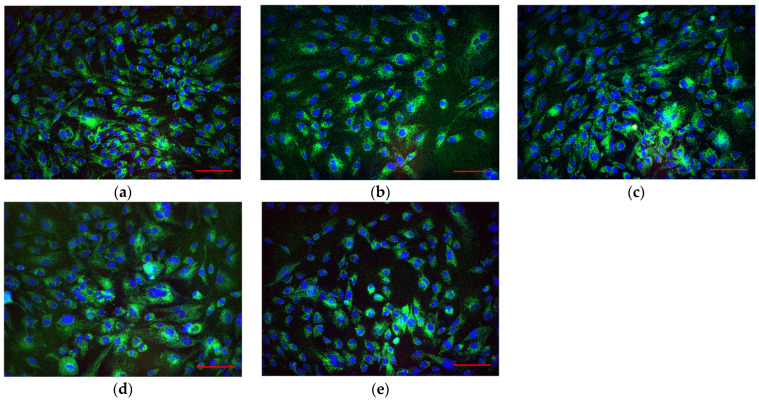
Representative microphotographs of HSF cell cultures after 72 h of in vitro cultivation. Control (**a**); in the presence of a sample fabricated from lithographic resin (PMMA) without CNPs (**b**); in the presence of samples fabricated from composite lithographic resin (PMMA) containing CNPs at concentrations of 0.001% (**c**), 0.01% (**d**), and 0.1% (**e**). The scale bar (red line) in the lower right corner corresponds to 10 µm.

The effect of photolithographic resin products on HSF mammalian cell culture was investigated (Figure 14). Microscopic examination revealed no significant differences between any of the studied cell groups. It was demonstrated that the cells adhere and spread with equal efficiency on both culture glass surfaces and fabricated samples with or without CNPs.

**Figure 15:**
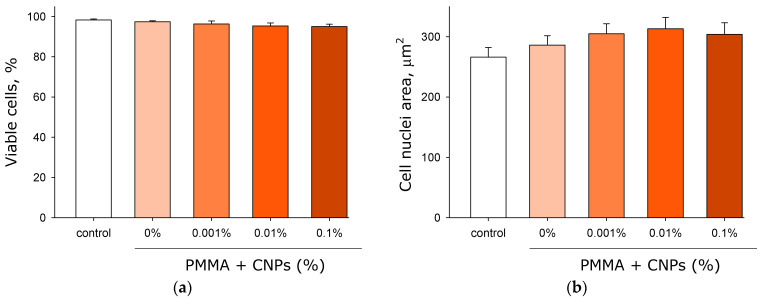
Effects of samples fabricated from photolithographic resin (PMMA) with and without CNPs on the growth and development of eukaryotic cells. Results of viability assessment of HSF cell cultures after 72 h of contact with the materials. Proportion of viable cells in cultures (**a**), nuclear area (**b**). Data are presented as the mean values ± standard deviation (*n* = 3).

Cell viability was assessed using fluorescence microscopy (Figure 15a). In all preparations, visual inspection generally revealed a small number of non-viable cells. Quantitative image analysis established that the proportion of viable cells exceeded 95% in all groups. It should be noted that no statistically significant differences were observed between any of the groups.

The degree of influence of the fabricated samples with or without CNPs on nuclear size was investigated (Figure 15b). It was shown that nuclear size did not differ among any of the experimental groups.

## 4. Discussion

Numerous methods for nanoparticle synthesis exist today [49]. Laser-based techniques, such as laser ablation and laser fragmentation, unlike most other methods, do not require chemical reagents and allow for precise control over key nanoparticle characteristics [50]. Using the laser ablation method, nanoparticles composed of carbon atoms have previously been synthesized, predominantly amorphous carbon [51], less frequently graphite nanoparticles [52], single-wall carbon nanotubes [53], fullerenes [54], and graphene [55]. In this work, complex-shaped carbon nanoparticles were obtained (Figure 1), and Raman spectroscopy data indicate that they consist of amorphous carbon (Figure 3).

A protocol for incorporating CNPs into the PMMA-based resin was developed. Samples of complex geometry with a maximum achievable resolution of 15 µm can be printed from the photolithographic resin containing CNPs at concentrations up to 0.1% (Figure 4). The photolithographic resin we use enables the production of optically transparent products. When using MSLA printing technology, researchers often encounter various surface defects, including optical ones, in the final products when nanoparticles are added to the resin [56]. It is also known that the presence of nanoscale additives in the resin for MSLA printing can complicate the post-processing of products [57], including surface polishing [58]. The PMMA-based composite resin with CNPs is amenable to polishing. After polishing samples with the highest CNP content (0.1%), a height variation of less than 10 nm over an area of 100 µm^2^ is observed (Figure 5).

Nanoparticles themselves rarely cause polishing issues, as their size is comparable to the smallest surface height variations [59]. A common problem is the uneven distribution of nanoparticles and their aggregation within the polymer [60]. According to current literature, nanoparticles are very often unevenly distributed in polymers [61,62,63,64]. In the samples we fabricated, no optical defects were observed under transmitted light illumination. When examined in reflection mode, the samples reflected objects without distortion and with sharp boundaries. Nevertheless, microscopy based on modulation-interference principles demonstrated the existence of regions with numerically similar phase shifts within the samples (Figure 6). The laser source of the modulation-interference microscope has a wavelength of 405 nm. The refractive index of amorphous carbon at this wavelength is 2.54 [65], while the refractive index of the PMMA resin used is 1.50 [66]. The refractive index values of the two materials differ substantially (by approximately 70%). This significant difference allows for clear distinction between CNPs and the PMMA resin. It should be noted that while regions with numerically close phase shifts were detected in the samples, no extended regions or regions of macroscopic size were found.

In lithographic photoprinting, the resin solidifies under light exposure through the polymerization of its components [67]. However, not all components participate in the polymerization—this is one of the key challenges of both MSLA technology and photolithography in general [68]. Monomers that do not participate in the polymerization ultimately remain within the fabricated structures, negatively affecting the material’s mechanical properties and the product’s strength characteristics [69]. Moreover, these unreacted resin monomers can leach out from the manufactured objects, and they are often toxic to mammalian cells [70]. The incorporation of CNPs into PMMA enhances the degree of resin polymerization upon exposure to ultraviolet radiation during photoprinting (Figure 7). Any allotropic form of carbon is oxidized by ultraviolet radiation in the presence of molecular oxygen. We assume that keto groups can form on the surface of CNPs under such conditions. Keto groups allow CNPs to react with other carbon-containing compounds under the influence of UV radiation. If the carbon-containing compound is PMMA, then the degree of its polymerization should increase. Currently, similar processes are shown for the allotropic form of carbon, graphite [55,56,57,58]. It should be noted that the addition of nanoparticles to the resin leads to an increase in the mechanical (Figure 8a) characteristics, which also indirectly confirms the involvement of nanoparticles in the resin polymerization process. When heated, the resin without nanoparticles showed a significantly greater weight loss compared to the resin with nanoparticles (Figure 8b), which also argues that the polymerization efficiency is higher in the presence of nanoparticles. The observed increase in the degree of resin polymerization during photo printing in the presence of ULF is important both for improving the production process and for improving the properties of the final products.

It has been established that CNPs exhibit significant antioxidant properties (Figure 9a,b). It is known that many allotropic forms of carbon demonstrate antioxidant properties; for example, the antioxidant properties of C_60_ fullerenes [71], graphene oxide [72], and carbon nanotubes [73] are well-documented. There are reports in the literature that amorphous carbon exhibits antioxidant properties in chemical systems [74,75], but these properties cannot be described as substantial or strong. In general, the antioxidant properties of carbon materials are associated with their system of conjugated double bonds [76]. Due to this property, fullerenes are even referred to as “radical sponges” [77]. An increase in reactive oxygen species (ROS) concentration in living systems is typically associated with the development of oxidative stress [78]. During oxidative stress, oxidative damage to proteins [79], nucleic acids [80], and lipids [81] is observed. Oxidative stress can even lead to loss of viability [82]. It is known that oxidative stress and the level of biomacromolecule damage can be reduced using antioxidants [83]. This work demonstrates that in the presence of objects made from photolithographic resin with CNPs, there is less intensive formation of the oxidative damage marker 8-oxoguanine in DNA (Figure 10a) and of long-lived reactive protein species (Figure 10b). Previous studies have shown that free antioxidant amino acids effectively prevent the formation of 8-oxoguanine in DNA under heat stress [43]. It is known that long-lived reactive protein species are generated, among other conditions, during hyperthermia and can themselves serve as a source of ROS in the environment [84]. Low-molecular-weight antioxidants such as guanosine and inosine can effectively eliminate long-lived reactive protein species [85], reducing the intensity of oxidative stress [86]. It should be noted that an increase in ROS concentration within certain limits in the environment can also have positive effects on living organisms [87].

Using laser breakdown, the possibility of nanoparticle release from polymer products was studied (Figure 11). Samples of material containing and not containing nanoparticles were placed in water for 3 days. It is known that a laser pulse with an energy of 2 mJ at full focus cannot cause optical breakdown of pure water. Breakdown is possible only with the appearance of seeds in the water. Nanoparticles of any nature can serve as seeds [34]. It was shown that the probability of optical breakdown does not change significantly when studying several million events. The sensitivity of the method is approximately 100–1000 nanoparticles per ml of liquid. Two conclusions immediately follow from this. First, nanoparticles do not release from the polymer, and if they do, it is lower than the detection level. Second, PMMA does not dissolve in water and does not form nanoparticles or any optical inhomogeneities capable of acting as seeds.

It has been shown that photolithographic resins with CNPs exert an inhibitory effect on the growth and development of *E. coli* bacteria (Figure 12). Moreover, the presence of CNPs in the samples causes a loss of bacterial viability (Figure 13). The antibacterial properties of carbon-based nanoparticles are thought to involve several alternative mechanisms of action [88]. Contact-mediated killing is considered the primary antibacterial mechanism for most nanoparticles, based on the interaction of CNPs with the bacterial membrane [89]. Carbon nanoparticles possess a localized surface charge and effectively interact with the bacterial cell wall, disrupting membrane transport [90]. Within bacterial cells, carbon nanoparticles can induce oxidative stress, partly due to their photocatalytic activity [91]. Despite their pronounced antibacterial properties, samples made from photolithographic resins with CNPs did not affect the growth and development of mammalian cells (Figure 14 and Figure 15). Furthermore, the cells achieved complete spreading on the surface of the resin samples containing CNPs. The photolithographic resin used in this work, without nanoadditives, lacked cytotoxicity—unlike many other PMMA-based resins. The addition of CNPs to the resin did not alter the viability of the studied cell culture, even though it has been previously demonstrated that various amorphous carbon nanoparticles, despite their unique properties, are toxic to mammalian cells [92]. The obtained PMMA-based resins with CNPs may potentially serve as a foundation for non-toxic materials in biomedicine and pharmacology.

## 5. Conclusions

Carbon nanoparticles (CNPs) of complex morphology with an average size of approximately 100 nm were obtained. Raman spectroscopy data indicate that the nanoparticles consist predominantly of amorphous carbon. A protocol for incorporating CNPs into a photolithographic resin at concentrations of up to 0.1% was developed. Samples of complex geometry were printed from the resulting resins with a maximum achievable resolution of 50 µm. The fabricated structures are amenable to polishing. After polishing of the samples with the highest CNP content (0.1%), a height variation of less than 10 nm over an area of 100 µm^2^ was observed. The CNPs are distributed relatively uniformly within the samples; however, regions differing in the phase shift in visible light at a wavelength of 405 nm were detected. It was established that the incorporation of CNPs increases the degree of polymerization of the resin during photoprinting. The CNPs were shown to exhibit significant antioxidant properties, substantially preventing the formation of oxidative damage products such as 8-oxoguanine in DNA and long-lived reactive protein species. Furthermore, photolithographic resins with CNPs were found to inhibit bacterial growth and development, and even cause a loss of bacterial viability. Despite the pronounced antibacterial properties, the samples made from CNP-containing photolithographic resins did not affect the growth and development of mammalian cells. We hope that the developed material will find its application for Counteracting Healthcare-Associated Infections.

## Data Availability

The raw data supporting the conclusions of this article will be made available by the authors on request.
